# Integrative analysis of TP73 profile prognostic significance in WHO grade II/III glioma

**DOI:** 10.1002/cam4.4016

**Published:** 2021-06-13

**Authors:** Yanming Chen, Ye Wang, Qiheng He, Wen Wang, Tan Zhang, Zhongyong Wang, Jun Dong, Qing Lan, Jizong Zhao

**Affiliations:** ^1^ Department of Neurosurgery The Second Affiliated Hospital of Soochow University Suzhou China; ^2^ China National Clinical Research Center for Neurological Diseases Beijing China; ^3^ Heath Management Center The Second Affiliated Hospital of Soochow University Suzhou China; ^4^ Department of Neurosurgery Beijing Tiantan Hospital Capital Medical University Beijing China

**Keywords:** biomarkers, cancers risk factors, methylation, prognosis, survival

## Abstract

Due to the extremely intrinsic heterogeneity among glioma patients, the outcomes of these patients are tremendously different. Therefore, the exploitation of novel biomarker classification of glioma is vitally important for deep insight into the essence and predicting the prognosis of glioma. We aim to analyze the correlation between TP73 mRNA expression, DNA methylated alteration and the prognosis of WHO grade II/III glioma, utilizing bioinformatics to evaluate its significance as a risk‐factor in predicting the prognosis of these glioma patients. The analysis found that TP73 expression was positively correlated with the grade of glioma, and showed a strong correlation with glioma molecular classification, which revealed significantly higher TP73 expression in IDH‐wildtype than in IDH‐mutant subtype of WHO grade II/III glioma. Cox regression analysis indicated that high expression of TP73 shared an independent high‐risk factor impacting the prognosis of WHO grade II/III glioma. We discovered 8 DNA promoter methylation sites with prognostic significance, which were negatively associated with TP73 expression, and positively associated with beneficial overall survival (OS) and progression‐free survival (PFS). Integrating with four independent glioma datasets, subsequent Meta‐analysis verified that low expression of TP73 was closely related to favorable OS, especially in IDH‐mutant subtype. Moreover, we found that 1p/19q^Codel^/TP73^low^ subgroup shared the most favorable OS, 1p/19q^Non−codel^/TP73^high^ subgroup suffered the worst OS. Meanwhile, the enrichment analysis of TP73‐related differential mRNAs demonstrated that TP73 aberration in WHO grade II/III glioma might be closely related to cell cycle and P53 signaling pathways. Finally, TP73 expression of 53 glioma specimens was measured by qRT‐PCR, which was consistent with the previous analytical result, and TP73 high‐expression subgroup suffered worse PFS than TP73 low‐expression subgroup. In summary, our funding supports that TP73 gene can perform as a reliable biomarker to evaluate the survival outcome of patients diagnosed with WHO grade II/III glioma.

## Graphical abstract

TP73 mRNA expression and DNA methylation profile can be a risk factor impacting the prognosis of WHO grade II/III glioma independently, or combining with current glioma molecular classification. Our work showed the significant potential of TP73 gene as a molecular characteristic for evaluating the malignancy and survival outcome of WHO grade II/III glioma.

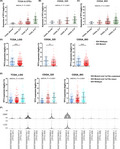

## 1 INTRODUCTION

Glioma represents the most common primary malignant tumor of the central nervous system.^1^ We general classify glioma into 4 grades, according to its histological feature in WHO Classification of Tumours of Central Nervous System.^2^, ^3^ Among them, grade I/II glioma has a relatively favorable prognosis and is defined as low‐grade gliomas (LGGs); while grade III/IV glioma always suffers a rapid progression and has a relatively poor prognosis, is classified as high‐grade gliomas (HGGs).^4^ Despite the considerable advance of neurosurgical technology,^5^ and adjuvant therapeutic approaches,^6^, ^7^ tumor therapeutic resistance and progression seem inevitable, especially for WHO grade IV glioblastoma multiforme (GBM), with a dismal median survival time of 14 months,^8^ and long‐term survival is rare.^9^ Unlike GBM, WHO grade II/III glioma, remaining approximately 45% of glial tumor,^10^ suffers a diverse survival outcome.^4^, ^11^ Hence, it's urgent to exploit novel prognostic significance biomarkers for such glioma.

TP73 maps to the region of chromosome 1p36. Interestingly, as a specific pathological type of glioma, oligodendroglioma frequently undergoes 1p/19q codeletion.^12^ 2016 WHO classification incorporate IDH mutation and 1p/19q codeletion status to further classified WHO II/III glioma into three molecular subtypes: IDH‐wildtype, IDH‐mutant and 1p/19q intact, and IDH‐mutant and 1p/19q codeleted.^3^ But 1p/19q codeletion pathogenetic implication remains incompletely clear. Those inspired us to disclose the role of TP73 participation in the pathogenesis of glioma. The TP73 gene encodes a protein of protein 73 (p73), which is a member of the p53 family.^13^ Unlike TP53, a powerful tumor suppressor gene, the function of TP73 remains controversial.^14^, ^15^ TP73 gene has two independent promoters and alternative splicing combinations, resulting in large numbers of transcript variants. In general, p73 contains two main isoforms of protein: Trans activating (TA) and deltaN (ΔN). TAp73 isoform exerts as p53‐like regulating cell cycle arrest, apoptosis and senescence.^13^ However, it has been reported that ΔNp73 more likely to involve the process of tumor progression.^16^ The above studies imply that the final functional destination of TP73 in different individuals is likely to depend on the final competition of different isoforms and their encoded products. Although the pathogenetic role of TP73 gene in neuroblastoma,^17^ hepatocellular carcinoma,^18^ and breast cancer^19^ has been reported, previously. So far, few studies focus on the association of TP73 alteration and prognosis significance of glioma,^14^, ^20^ the correlation between TP73 expression and 1p/19q codeletion, their integrated prognostic significance in WHO grade II/III glioma, additionally.

Mutations in TP73 gene are rare,^14^ so the expression level of its transcript variants depends more on transcriptional regulation. As a common epigenetic modification, DNA methylated modification plays a vital role in regulating gene expression at the transcriptional level.^21^ Hyper methylation of the promoter CpG islands has been etiologically associated with transcriptional inactivation of numbers of tumor suppressor genes in tumor progression.^22^ Moreover, DNA methylated alteration in tumor cells is often closely associated with anti‐tumor drug resistance.^23^ The demethylation of the MGMT in glioma leads to tumor cell resistance to alkylating agents such as temozolomide.^24^ Therefore, it is necessary to synthesize the TP73 mRNA expression and DNA methylation level when studying the biological role of TP73 in glioma and its prognostic significance.

It is necessary to make a prior statement that the cases in the LGG dataset of The Cancer Genome Atlas (TCGA) database consist of WHO grade II and III glioma, instead of the LGGs we mentioned above. To avoid unnecessary confusion, we use the grade of glioma in the following context. In this study, we first explored the correlation between TP73 mRNA expression and glioma classification; and analyzed the prognostic significance of TP73 mRNA expression and DNA methylation. After that, integrated with the four datasets containing WHO grade II/III glioma cases, we further verified the credibility of TP73 acted as an independent prognostic risk factor for grade II/III glioma. Meanwhile, TP73 mRNA expression profile and its prognostic prediction capacity were verified by a cohort of glioma specimens. Moreover, we utilized a combination of 1p/19q codeletion status and TP73 expression level to analyze the prognosis of WHO grade II/III glioma. Finally, we preliminarily predicted the possible functional mechanism of aberrant TP73 in glioma via TP73‐related differential mRNA in grade II/III glioma.

## 2 MATERIALS AND METHODS

### 2.1 Datasets preparation

We downloaded Next‐generation RNA sequencing data (Platform: Illumina) and clinical data of 529 glioma cases from TCGA (https://portal.gdc.cancer.gov/) database. Supplementary clinical data and DNA methylation data (Platform: Illumina Human Methylation 450) of the above cases were obtained from UCSC Xena (http://xena.ucsc.edu/) and GlioVis (http://gliovis.bioinfo.cnio.es/). Among them, there were 257 cases of WHO grade II glioma and 266 cases of grade III glioma. Downloaded three cohorts of data from Chinese Glioma Genome Atlas (CGGA) (http://www.cgga.org.cn/) database, CGGA_325 RNA sequencing data (Grade II: 98 cases; Grade III: 74 cases; Platform: Illumina HiSeq), CGGA_693 RNA sequencing data (Grade II: 172 cases; Grade III: 255 cases; Platform: Illumina HiSeq) and CGGA_301 mRNA array data (Grade II: 106 cases; Grade III: 53 cases; Platform: Agilent Whole Human Genome). Inclusion criteria: (1) Cases diagnosed with grade II or III glioma, definitely; (2) Cases with complete prognostic follow‐up data. A total of 1281 cases was included in this research. 286 glioma whole‐exome sequencing (WESeq) data (Platform: Illumina HiSeq 4000) were obtained from CGGA database.

### 2.2 Univariate and multivariate Cox regression analysis

TP73 gene mRNA expression and DNA methylation level data were combined with the corresponding clinical data of each case to perform survival analysis. Univariate and multivariate Cox proportional hazards regression analyses were utilized to evaluate the prognostic significance of TP73 mRNA expression and DNA methylation.

### 2.3 Meta‐analysis

A retrieved result from PubMed, Medline, and Web of Science databases, just one published research,^25^ which harbored a total of 12 cases of WHO grade II/III glioma, did not find any other research involving the TP73 and prognosis of WHO grade II or III gliomas. Therefore, we extracted 3 datasets of WHO grade II/III glioma cases from CGGA database; and integrated them with the LGG dataset of TCGA database. We utilized Meta‐analysis to evaluate the effect of TP73 gene expression to predict overall prognostic significance of patients diagnosed with WHO grade II or III glioma. Q test (I^2^ statistics) were utilized to assess the heterogeneity of 4 datasets. A random‐effects model was selected for combination if were obvious heterogeneity (I^2^ ≥ 50% or *p*‐valve <0.1), otherwise chose the fixed‐effects model. The "meta" package was applied in Meta‐analysis of the research.

### 2.4 Screening TP73‐related differentially expressed mRNA

Different grades of glioma were divided into high‐expression and low‐expression groups based on the median expression of TP73. The "limma" package was utilized to filter the differentially expressed mRNAs (DEmRNAs) between two groups, defined as TP73‐related differential genes. The filtering criteria followed as false discovery rate (FDR) <0.05, and the absolute value of log_2_FC (fold change) >0.6.

### 2.5 Gene ontology (GO) and kyoto encyclopedia of genes and genomes (KEGG) analysis of TP73‐related DEmRNAs

The “clusterProfiler” package was utilized for DEmRNAs functional annotation analysis. The filter criteria for GO and KEGG enrichment analysis were *p*‐value <0.05, and FDR <0.05.

### 2.6 Clinical specimens and quantitative real‐time PCR (qRT‐PCR)

All 53 glioma specimens of different grades were collected between February 2017 and October 2020. Total RNA was extracted from shredded glioma tissue with TRIzol (Invitrogen). Then, cDNA was synthesized with a First Strand cDNA Synthesis Kit (Thermo Scientific). qRT‐PCR was performed as previous report.^26^ Each sample was run in triplicate, and the relative mRNA expression was calculated using the formula 2^−ΔΔCt^ by the comparative ΔΔCt method. The primers used were as follows: TP73 (5’‐CCACCACTTTGAGGTCACTTT‐3’, 5’‐CTTCAAGAGCGGGGAGTACG‐3’); GAPDH (5’‐GAAGGTGAAGGTCGGAGTC‐3’, 5’‐GAAGATGGTGATGGGATTTC‐3’).

### 2.7 Statistical analysis

GraphPad Prism (version 6.0) or R (version 4.0.2) was used for data analysis. The mean value differences were compared by analysis of variance (ANOVA) in multiple groups. A Student's *t* test was used to identify the difference between two groups. Kaplan‐Meier curves and Log‐Rank test were utilized to evaluate prognostic outcomes. Time‐dependent receiver operating curve (ROC) analysis was used to assess the survival prognostic predictive accuracy. The χ^2^ test was utilized to identify significant differences between groups. Spearman correlation test was used to calculate the correlation between TP73 mRNA expression and DNA methylation. Gene enrichment analysis was validated by Gene set enrichment analysis (GSEA). A *p*‐value <0.05 suggested statistical significance.

## 3 RESULTS

### 3.1 Frequency spectrum analysis of TP73 gene mutations in glioma

Analyzing the WESeq data of 286 different grades of glioma in CGGA database, the results indicated that compared with the high mutation frequency of TP53 in glioma (131/286), the mutation frequency of TP73 in glioma was very low (2/286) (Figure S1A). The frequency of TP53 mutations tended to elevate with the grade of glioma (Figure S1B). It was suggested that the predominant cause for the abnormal functions of TP73 in glioma might not the alteration of genome sequence. Transcriptional and post‐transcriptional regulation of TP73 might play a critical role in exerting dysfunctions in glioma.

### 3.2 TP73 expression profile in different subtypes of glioma

**FIGURE 1:**
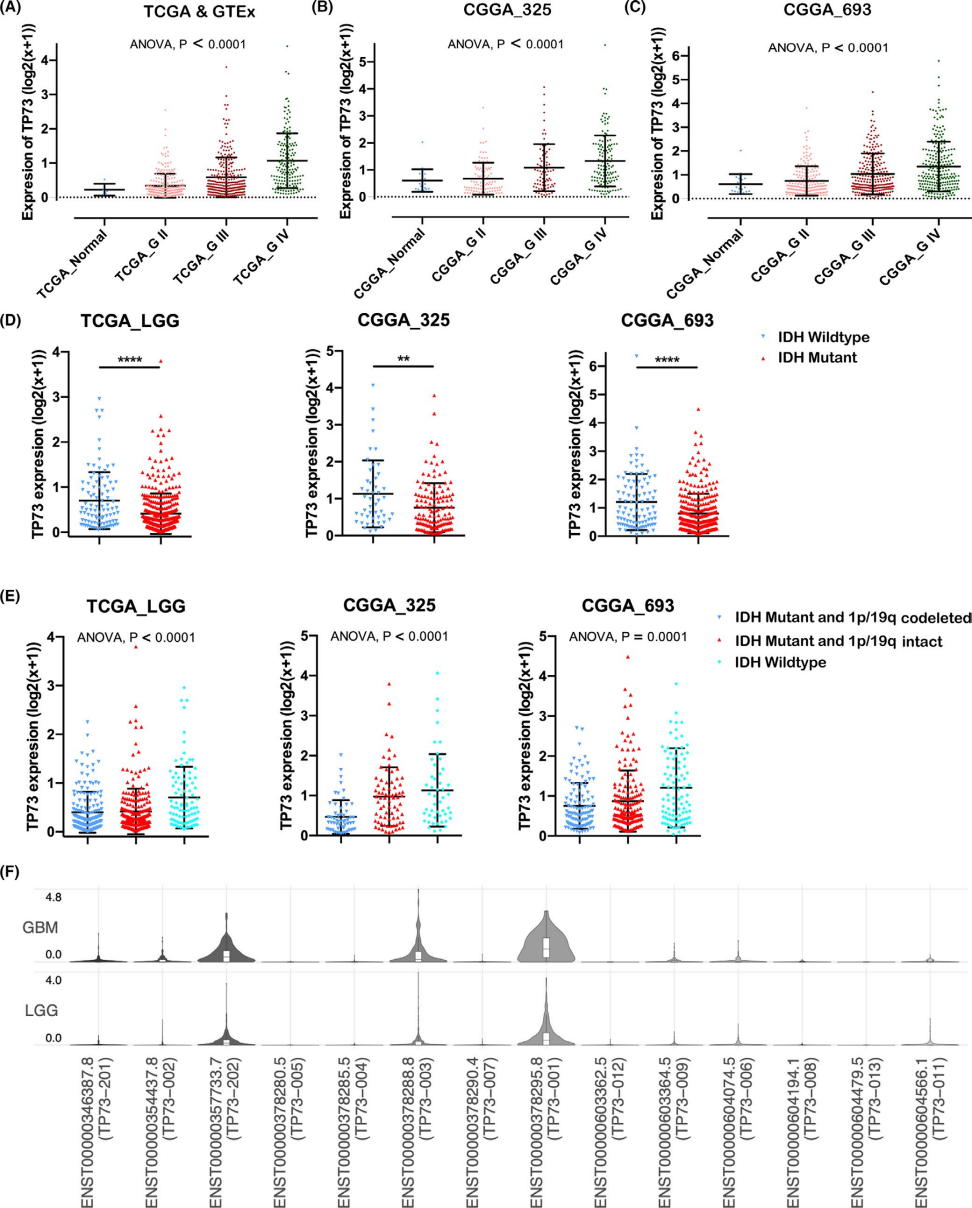
(A‐C) TP73 expression in different grades of glioma and normal cerebral tissues in TCGA, CGGA_693 and CGGA_693 datasets, respectively. The differences between groups were analyzed with one‐way ANOVA followed by Student–Newman–Keuls tests. (D) The difference of TP73 expression between IDH‐wildtype and IDH‐mutant subtypes in TCGA_LGG, CGGA_325 and CGGA_693 datasets, respectively. (E) The difference of TP73 expression among IDH‐mutant and 1p/19q codeleted, IDH‐mutant and 1p/19q intact, and IDH‐wildtype subtypes in TCGA_LGG, CGGA_325 and CGGA_693 datasets, respectively. (F) The expressive distribution of TP73 transcript isoforms in LGG and GBM datasets of TCGA database. **represent *p* < 0.01; ****represent *p* < 0.0001

There were 257 cases of grade II glioma in TCGA database, 266 cases of grade III glioma, 168 cases of grade IV GBM, and 5 normal cerebral samples. We found that TP73 mRNA was lower expressed in normal cerebral tissues, and TP73 expression was positively correlated with the grade of glioma, strikingly (*p* < 0.0001) (Figure 1A). We also observed the similar expression profile in CGGA_325 and CGGA_693 datasets of the CGGA database (*p* < 0.0001; *p* < 0.0001, respectively) (Figure 1B–C). Based on IDH mutation status classification of WHO grade II/III glioma, remarkable differences of TP73 expression between IDH‐mutant subtype and IDH‐wildtype subtype were also observed in WHO II/III glioma. The expression of TP73 in IDH‐wildtype subtype was significantly higher than that in IDH‐mutant subtype in TCGA_LGG, CGGA_325 and CGGA_693 datasets of WHO grade II/III glioma (*p* < 0.0001; *p* = 0.0029; *p* < 0.0001, respectively) (Figure 1D). Further analysis found that the expression of TP73 in IDH‐mutant combined with 1p/19q codeleted subtype was significantly lower than that of IDH‐wildtype subtype in the 3 cohorts of datasets (*p* < 0.0001; *p* < 0.0001; *p* = 0.0001, respectively) (Figure 1E).

**FIGURE 2:**
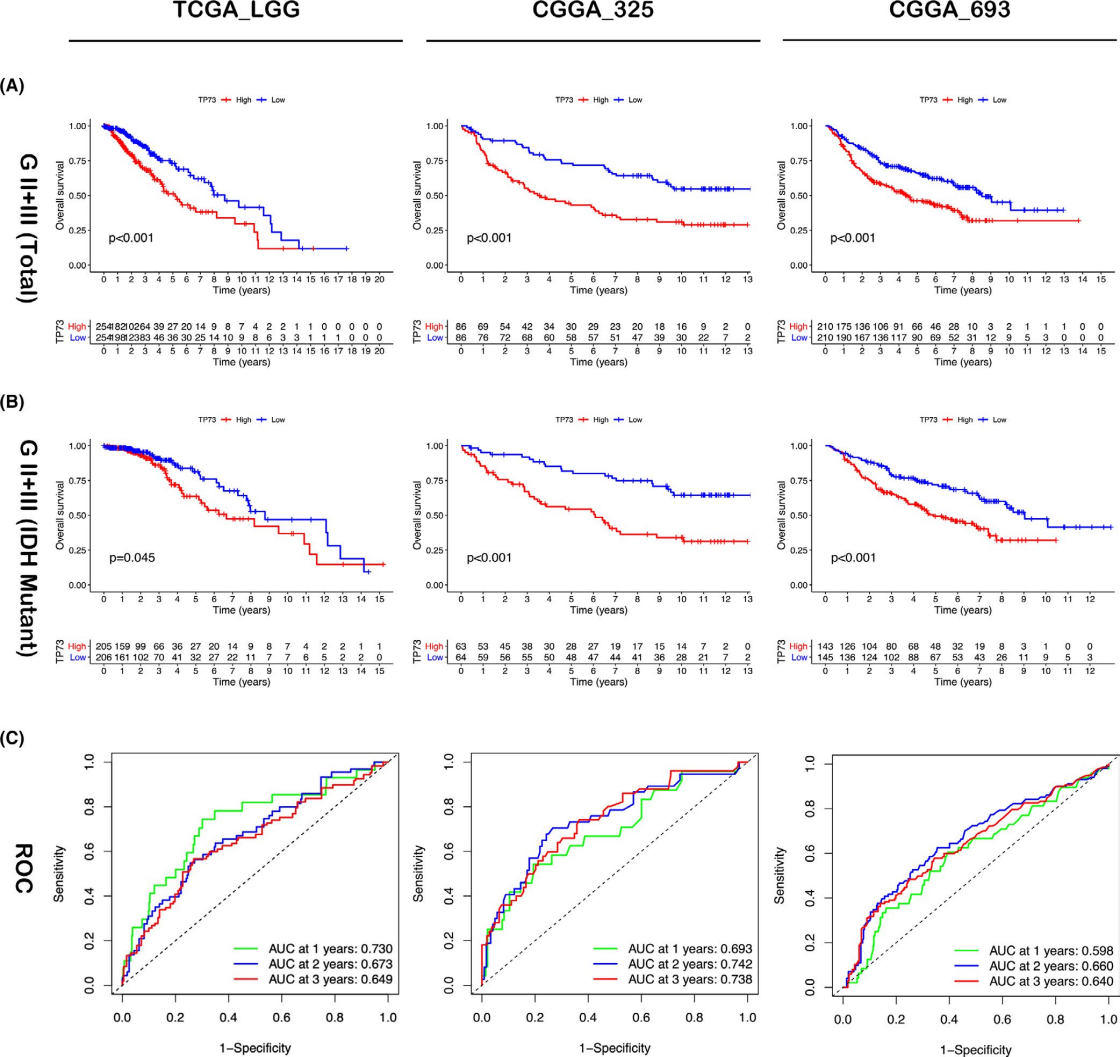
(A) The survival curves of WHO grade II/III glioma high‐expression and low‐expression subgroups in TCGA_LGG, CGGA_325 and CGGA_693 datasets. (B) The survival curves of IDH‐mutant subtype glioma high‐expression and low‐expression subgroups in TCGA_LGG, CGGA_325 and CGGA_693 datasets. (C) Area under the ROC curves of TP73 expression prognostic prediction for WHO grade II/III glioma within 1, 2 and 3 years in TCGA _LGG, CGGA_325 and CGGA_693 datasets, respectively

Moreover, the expression levels of each TP73 transcript isoforms were analyzed by GEPIA2 (http://gepia2.cancer‐pku.cn/#index), which indicated that two isoforms (TP73‐202 and TP73‐001, both of which were classified as TAp73 isoform) with the highest expression were significantly higher expressed in GBM dataset than in LGG dataset (Figure 1F). We further dichotomized the cases into high‐expression and low‐expression groups based on the median expression of TP73. Univariate Cox regression survival analysis demonstrated that the OS and PFS of the high‐expression group in LGG dataset of TCGA database were significantly worse than that of the low‐expression group (*p* < 0.001; *p* < 0.001, respectively) (Figure 2A, Figure S2A). We verified that the OS of high‐expression groups were also significantly worse than that of the low‐expression group in 2 cohorts of CGGA database (*p* < 0.001) (Figure 2A). Further distinguished WHO grade II and III glioma in TCGA_LGG dataset, the high‐expression subgroup in grade III glioma also showed worse OS and PFS than the low‐expression subgroup (*p* < 0.001; *p* < 0.001, respectively) (Figure S2C); while the OS and PFS of the low‐expression subgroup in the WHO II glioma were also better than the high‐expression subgroup, although the differences were not statistically significant (*p* = 0.341, *p* = 0.137) (Figure S2B). Interestingly, the OS of high‐expression group remained significantly worse than that of low‐expression group in IDH‐mutant subtype (*p* = 0.045; *p* < 0.001; *p* < 0.001, respectively) (Figure 2B), while the trend of OS between high‐ and low‐expression groups was not so significant in IDH‐wildtype subtype (*p* = 0.163; *p* = 0.228; *p* = 0.001, respectively) (Figure S2D). Moreover, ROC curves indicated that TP73 expression could predict the OS of 1‐year, 2‐year and 3‐years with considerable predictability in different cohorts of WHO grade II/III glioma (Figure 2C, Figure S2B–C).

### 3.3 Independent prognostic analysis of clinical phenotype together with TP73 expression

**FIGURE 3:**
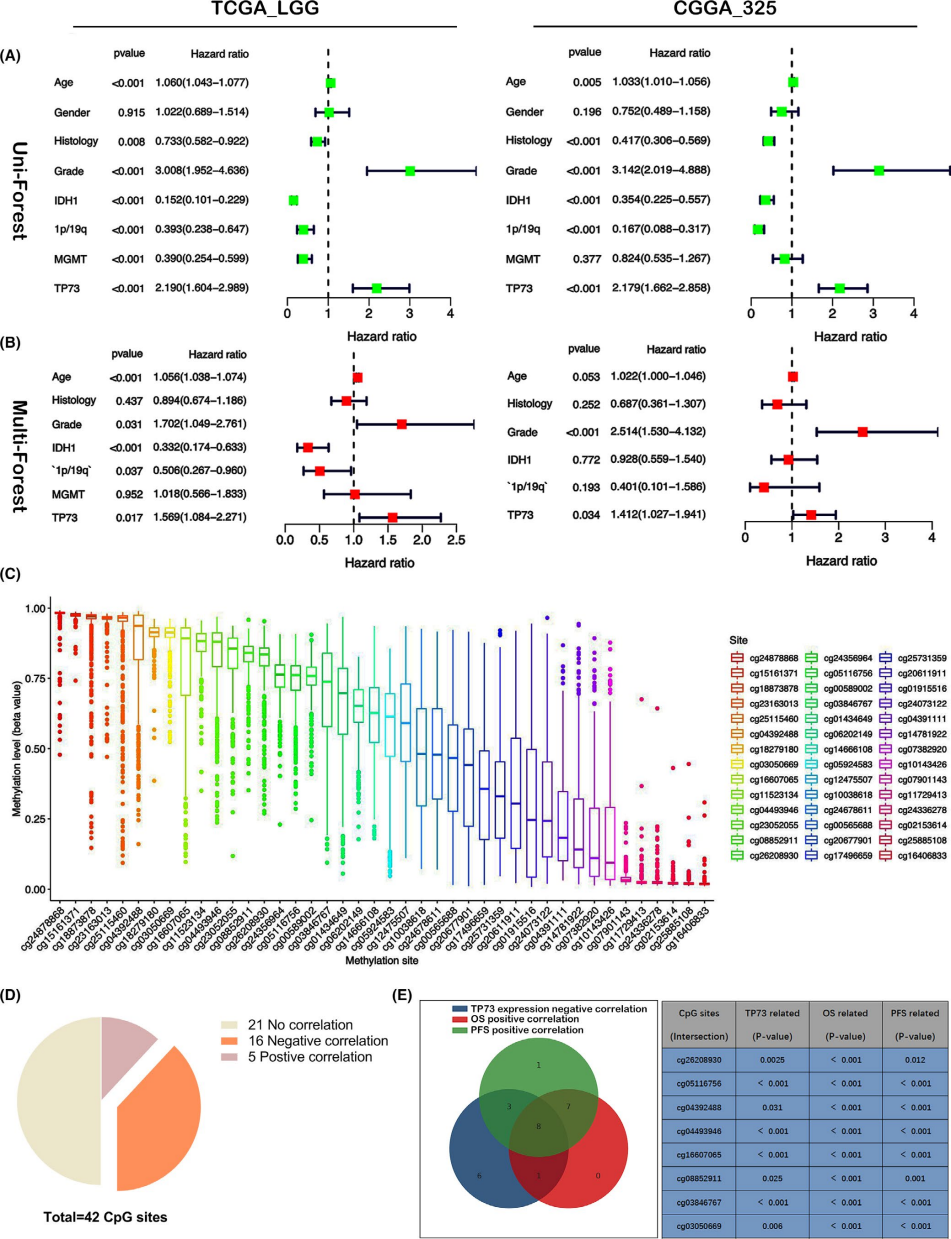
(A) Univariate Cox regression analysis presents a forest plot for TP73 expression and clinical phenotypes in TCGA_LGG and CGGA_325 datasets, respectively. (B) Multivariate Cox regression analysis presents a forest plot for TP73 expression and clinical phenotypes in TCGA_LGG and CGGA_325 datasets, respectively. (C) The distribution of 42 CpG islands methylation sites of TP73 gene in WHO grade II/III glioma. (D) The methylation levels of 16 CpG islands methylation sites were significantly positively correlated with the OS, 5 of which were significantly negatively correlated with the OS. (E) Venn diagram revealed that 8 CpG methylation sites were negatively correlated with TP73 expression and positively correlated with patient OS and PFS

A univariate Cox regression analysis of TP73 expression together with patient age, tumor grade, gender, IDH mutation status, 1p/19q codeletion status, MGMT promoter methylation status, and histopathological phenotype, in 3 WHO grade II/III glioma datasets, found that TP73 expression, tumor grade, IDH mutation status, 1p/19q codeletion status and histopathological phenotype always showed a significant correlation with survival prognosis (*p* < 0.05), and could be performed as independent prognostic factors (Figure 3A, Figure S3A). Moreover, the selected prognostic factors from univariate Cox regression analysis were further analyzed by multivariate Cox regression, which also indicated that TP73 expression performed as an independent prognostic factor (Figure 3B, Figure S3B), and the HR values of TP73 expression as an independent prognostic factor were 1.569 (95%CI, 1.084–2.271), 1.412 (95%CI, 1.027–1.941), and 1.268 (95%CI, 1.056–1.522) in TCGA_LGG, CGGA_325 and CGGA_693 datasets, respectively (*p* = 0.017, *p* = 0.034, and *p* = 0.011, respectively) (Figure 3B, Figure S3B). Hence, we are confident that high expression of TP73 is an independent high‐risk factor impacting the prognosis of WHO grade II/III glioma.

### 3.4 TP73 gene methylation profile of different subtypes glioma

Aberrant DNA methylation of TP73 has been observed in previous study,^27^ and there were large numbers of methylation sites in the promoter CpG islands of TP73 gene. By analyzing the LGG methylation dataset of TCGA database, 42 CpG sites were detected in the promoter region of TP73 gene, and the level of methylation at different methylation sites was exhibited in Figure 3C. A slight difference in TP73 methylation among different subtypes (Figure S4A–D). Among the 42 methylation sites, 21 methylation sites significantly correlated with survival time (*p* < 0.05), 16 methylation sites showed a positive correlation between the level of methylation and the better OS, and 5 methylation sites showed negative correlation (Figure 3D, Figure S5A). Further analysis revealed that 8 of the 16 methylation sites were significantly positively related to the better OS and PFS of patients diagnosed with grade II/III glioma (Figure 3E, Figure S5B–C). Meanwhile, the methylation level of those sites was negatively related to the expression of TP73 (Figure S5A).

### 3.5 TP73 gene expression and methylation related clinical phenotypes analysis

To further analyze the correlation between TP73 gene and clinical phenotypes, the LGG dataset of TCGA database was dichotomized into high‐expression and low‐expression subgroups, based on the level of TP73 mRNA expression and DNA methylation. A χ^2^ test was utilized to evaluate the correlation between TP73 expression and clinical phenotypes. As exhibited in Table 1, tumor grade, histopathological classification, IDH status, and molecular classification were remarkably correlated with TP73 expression; while the clinical phenotypes of patients’ age, gender, MGMT promoter methylation status, TERT promoter and ATRX mutation status showed no significant association with TP73 expression (Table 1). As to TP73 methylation, histopathological classification, molecular classification, IDH status, 1p/19q codeletion and MGMT promoter methylation status were significantly correlated with the TP73 methylation level (Table S1).

**TABLE 1:** The correlation analysis between TP73 expression and clinical phenotypes with χ^2^ test

Covariates	Clinical Features		TP73 Expression		*p*‐value
	Sub‐group	Percentage	High	Low	
Age	<=65	421 (93.35%)	206 (91.56%)	215 (95.13%)	0.1818
	>65	30 (6.65%)	19 (8.44%)	11 (4.87%)	
Gender	Female	199 (44.12%)	93 (41.33%)	106 (46.9%)	0.273
	Male	252 (55.88%)	132 (58.67%)	120 (53.1%)	
Histology	A	167 (37.03%)	99 (44%)	68 (30.09%)	0.0085
	OA	111 (24.61%)	51 (22.67%)	60 (26.55%)	
	O	173 (38.36%)	75 (33.33%)	98 (43.36%)	
Grade	II	212 (47.01%)	79 (35.11%)	133 (58.85%)	<0.0001
	III	234 (51.88%)	146 (64.89%)	88 (38.94%)	
	Unknown	5 (1.11%)	0 (0%)	5 (2.21%)	
IDH Status	Mut	363 (80.49%)	167 (74.22%)	196 (86.73%)	0.0006
	Unknown	2 (0.44%)	0 (0%)	2 (0.88%)	
	WT	86 (19.07%)	58 (25.78%)	28 (12.39%)	
1p/19q Status	Codel	151 (33.48%)	66 (29.33%)	85 (37.61%)	0.078
	Non‐Codel	300 (66.52%)	159 (70.67%)	141 (62.39%)	
Molecular Classification	IDH‐Mut/Codel	151 (33.48%)	66 (29.33%)	85 (37.61%)	0.0013
	IDH‐Mut/Non‐codel	212 (47.01%)	101 (44.89%)	111 (49.12%)	
	IDH‐WT	86 (19.07%)	58 (25.78%)	28 (12.39%)	
MGMT Promoter	Methylated	372 (82.48%)	182 (80.89%)	190 (84.07%)	0.4443
	Unmethylated	79 (17.52%)	43 (19.11%)	36 (15.93%)	
TERT Promoter	Mut	130 (28.82%)	65 (28.89%)	65 (28.76%)	1
	Unknown	169 (37.47%)	84 (37.33%)	85 (37.61%)	
	WT	152 (33.7%)	76 (33.78%)	76 (33.63%)	
ATRX Status	Mut	165 (36.59%)	79 (35.11%)	86 (38.05%)	0.5331
	Unknown	2 (0.44%)	0 (0%)	2 (0.88%)	
	WT	284 (62.97%)	146 (64.89%)	138 (61.06%)	

Abbreviations: A, Astrocytoma; Codel, Codeletion; Mut, Mutant; O, Oligodendroglioma; OA, Oligo astrocytoma; WT, Wildtype.

### 3.6 Meta‐analysis evaluated the value of TP73 expression in prognostic prediction

**FIGURE 4:**
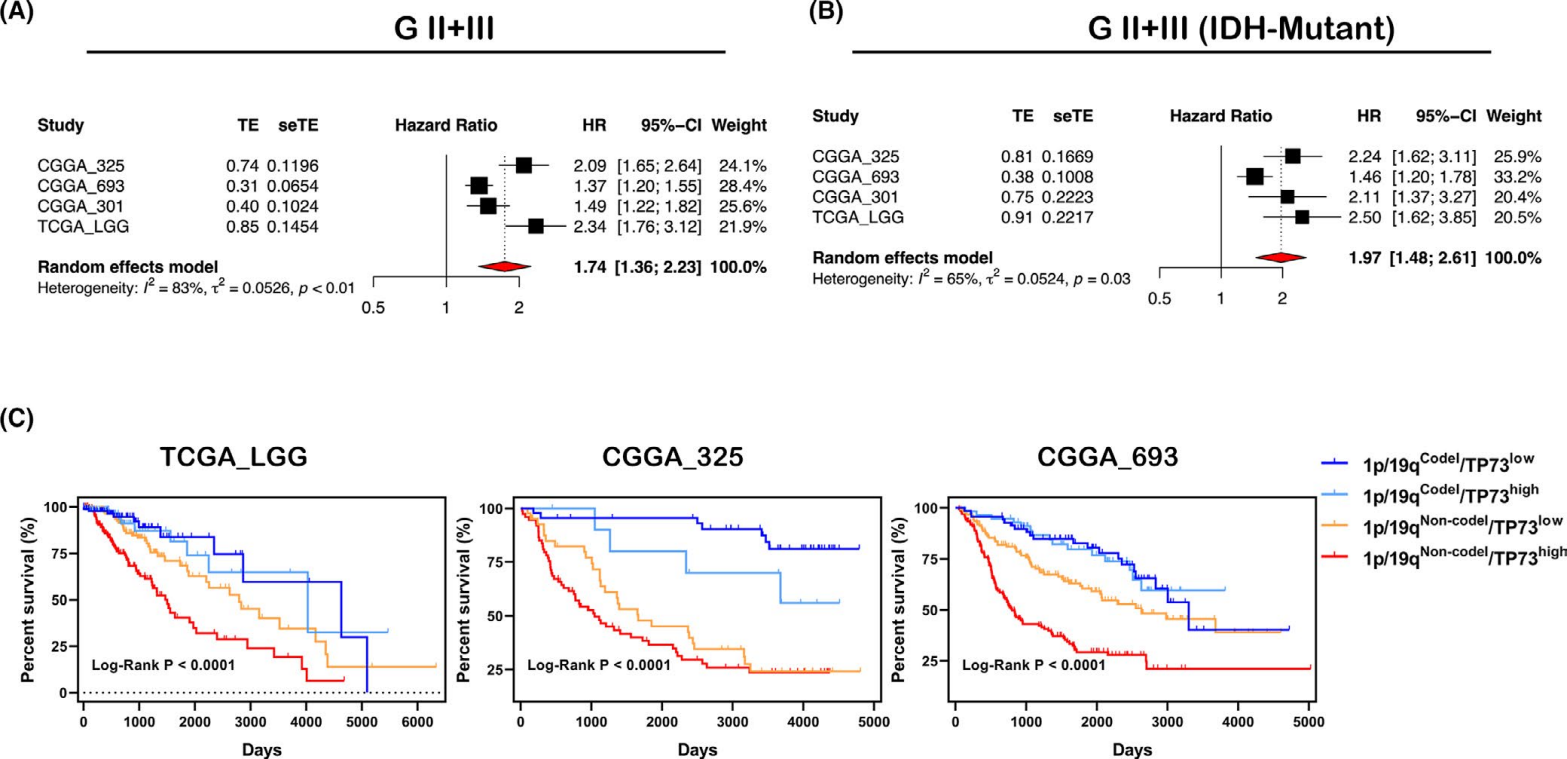
(A) Random forest plot of TP73 expression prognostic risk in WHO grade II/III glioma from 4 datasets with Meta‐analysis. (B) Random forest plot of TP73 expression prognostic risk in IDH‐mutant subtype from 4 datasets with Meta‐analysis. (C) Survival curves of patients classified with TP73 expression and 1p/19q codeletion status in TCGA_LGG, CGGA_325 and CGGA_693 datasets, respectively

Three datasets from CGGA database, combining with the LGG dataset in TCGA database, were included for Meta‐analysis. The prognostic Meta‐analysis of the above four datasets revealed that high expression of TP73 performed as a high‐risk factor impacting the survival of WHO grade II/III glioma, with HR (95%‐CI) values were 2.09 (1.65, 2.64), 1.37 (1.20, 1.55), 1.49 (1.22, 1.82) and 2.34 (1.76, 3.12) in CGGA_325, CGGA_693, CGGA_301 mRNA array and TCGA_LGG datasets, respectively (Figure 4A).

Based on the classification of IDH mutation status, followed prognostic Meta‐analysis indicated that high expression of TP73 still showed a strong high‐risk factor in IDH‐mutant WHO grade II/III glioma (Figure 4B); while TP73 high expression as a factor impacting survival prognosis was not so strong in IDH‐wildtype subtype (Figure S6A). In summary, TP73 expression has remarkable significance for the prognostic evaluation of WHO grade II/III glioma, especially for IDH‐mutant subtype WHO grade II/III glioma.

### 3.7 Correlation analysis of TP73 expression and glioma molecular classification

Because of the special genomic location of TP73 gene and the 1p/19q codeletion characteristic of oligodendroglioma, we further analyzed the impact of the IDH mutation and 1p/19q codeletion status on TP73 expression. WHO grade II/III glioma with IDH mutation and 1p/19q codeletion status in CGGA_325, CGGA_693 and CGGA_301 mRNA array datasets were enrolled in this research. We implemented χ^2^ test to analyze the correlation between 3 subtypes of glioma molecular classification and TP73 expression in 3 datasets of CCGA database. As shown in Table 2, the expression of TP73 were strongly correlated with glioma molecular classification of WHO grade II/III glioma in CGGA_325, CGGA_693 and CGGA_301 datasets (*p* < 0.0001; *p* = 0.0003; *p* = 0.0369, respectively). Besides, our analytical results reconfirmed that TP73 expression was strongly related to tumor grade (*p* < 0.05) (Table 2). We further distinguished CGGA_325 and CGGA_693 datasets by tumor grade, and the correlation analysis found that TP73 expression was significantly correlated with the status of 1p/19q codeletion regardless of tumor grade (*p* < 0.05) (Table S2), except for CGGA_693 grade II subgroup (*p* = 0.8715).

**TABLE 2:** The correlation analysis between TP73 expression and tumor classifications in CGGA database

Datasets	Covariates	Clinical features		TP73 expression		*p*‐value
		Sub‐group	Percentage	High	Low	
CGGA_325	Molecular classification	Mut/Codel	57 (31.67%)	13 (14.44%)	44 (48.89%)	<0.0001
		Mut/Non‐codel	75 (41.67%)	47 (52.22%)	28 (31.11%)	
		WT	48 (26.67%)	30 (33.33%)	18 (20%)	
	Grade	II	102 (56.67%)	41 (45.56%)	61 (67.78%)	0.0043
		III	78 (43.33%)	49 (54.44%)	29 (32.22%)	
CGGA_693	Molecular classification	Mut/Codel	109 (30.03%)	47 (26.26%)	62 (33.7%)	0.0003
		Mut/Non‐codel	171 (47.11%)	75 (41.9%)	96 (52.17%)	
		WT	83 (22.87%)	57 (31.84%)	26 (14.13%)	
	Grade	II	154 (42.42%)	62 (34.64%)	92 (50%)	0.0043
		III	209 (57.58%)	117 (65.36%)	92 (50%)	
CGGA_301	Molecular classification	Mut/Codel	16 (30.19%)	4 (15.38%)	12 (44.44%)	0.0369
		Mut/Non‐codel	23 (43.4%)	12 (46.15%)	11 (40.74%)	
		WT	14 (26.42%)	10 (38.46%)	4 (14.81%)	
	Grade	II	31 (58.49%)	11 (42.31%)	20 (74.07%)	0.0387
		III	22 (41.51%)	15 (57.69%)	7 (25.93%)	

Abbreviations: Codel, 1p/19q Codeletion; Mut, IDH‐mutant; Non‐Codel, 1p/19q Non‐Codeletion; WT, IDH‐wildtype.

Additionally, we classified the patients based on TP73 expression and 1p/19q codeletion status. The survival analysis utilizing Kaplan‐Meier curves revealed that patients in 1p/19q^Non−codel^/TP73^high^ subgroup suffered the worst OS, and 1p/19q^Non−codel^/TP73^low^ subgroup showed significantly favorable OS than the former subgroup in 3 datasets (Figure 4C). Moreover, 1p/19q^Codel^/TP73^low^ subgroup shared the favorite OS in CGGA_325 dataset (Figure 4C), while there was no significant difference between 1p/19q^Codel^/TP73^low^ subgroup and 1p/19q^Codel^/TP73^high^ subgroup in TCGA_LGG and CGGA_693 datasets (Figure 4C). Consistent with previous reports,^28^ our study revealed that the OS of 1p/19q Codeleted group was significantly better than that of 1p/19q Non‐codeleted group (Figure 4C, Figure S6C). Briefly, based on 1p/19q codeletion status, we included the variable of TP73 expression. The combination of two variables strongly strengthened the prognostic predictability of WHO grade II/III glioma, especially in 1p/19q intact subtype.

### 3.8 Enrichment analysis of TP73‐related differential mRNAs

**FIGURE 5:**
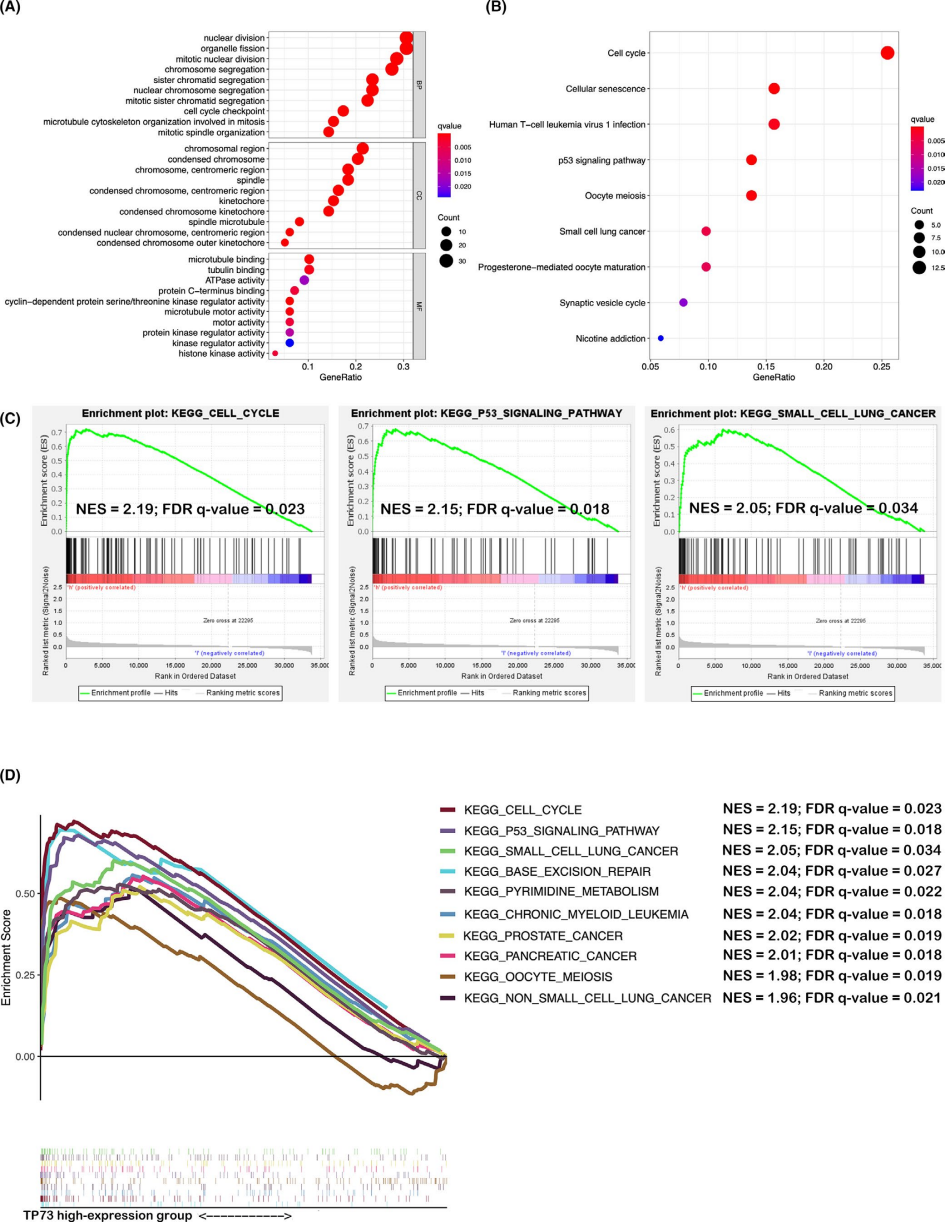
GO and KEGG analysis of TP73‐related DEmRNAs. (A) GO functional enrichment analysis; (B) KEGG pathway enrichment analysis. (C) GSEA indicated significant upregulation of cell cycle, P53 and small cell lung cancer pathway in the high‐expression group. (D) The top 10 enriched pathways in TP73 high‐expression group analyzed by GSEA. Abbreviations: NES, Normalized Enrichment Score

Finally, to better insight the functional role of TP73 gene in the occurrence and progression of glioma, we exploited the LGG dataset of TCGA database. 100 TP73‐related differentially expressed protein‐coding mRNAs were screened out (Table S3). The above 100 TP73‐related DEmRNAs were performed GO functional and KEGG pathway enrichment analysis, GO enrichment analysis showed top 10 terms of biological process, cellular component and molecular function, which indicated that TP73 might involve in mitotic nuclear division, chromosome segregation, and cell cycle checkpoint process (Figure 5A); while KEGG enrichment analysis implicated that aberrant TP73 might involves in the pathway of cell cycle, cellular senescence, and p53 signaling pathway (Figure 5B). Of note, TP73‐related DEmRNAs strongly involved in glial cell cycle arrest and senescence, which were all associated with glial cell malignant progression. GSEA analysis results were consistent with KEGG enrichment analysis. Cell cycle and P53 signaling pathway were the most significant upregulated pathways in TP73 high‐expression subgroup (Figure 5C). The top 10 most significantly upregulated pathways in TP73 high‐expression subgroup were majority associated with tumorigenesis (Figure 5D).

### 3.9 TP73 profile analysis from clinical specimens

**FIGURE 6:**
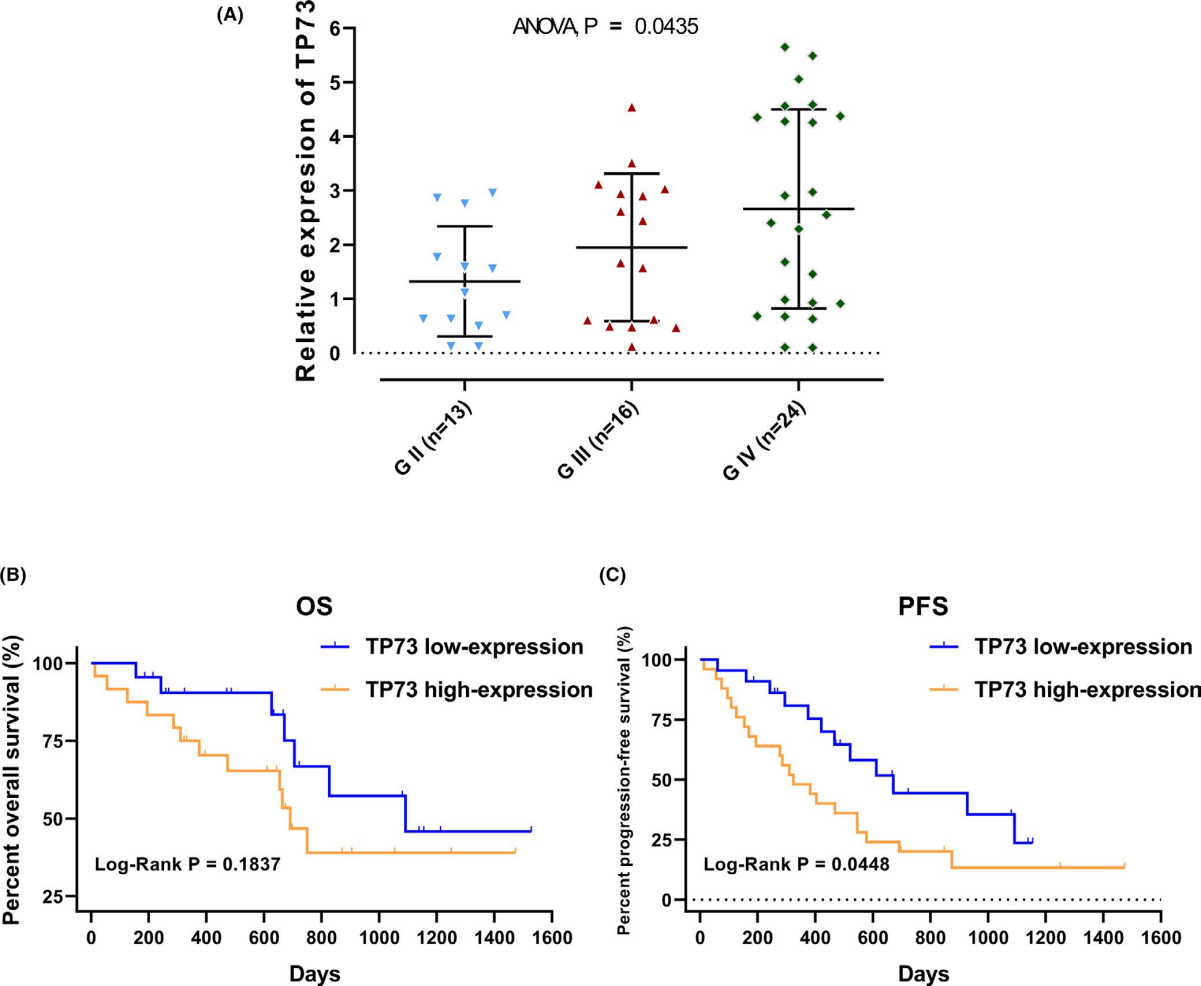
(A) The expression of TP73 in 53 glioma specimens with different WHO grades. (B‐C) Kaplan‐Meier analysis of OS and PFS according to high and low TP73 expression

We found that TP73 was differentially expressed in different types of glioma from the public databases of glioma. The expression level of TP73 was significantly associated with the pathological grade, IDH mutation status, 1p deletion status and survival times. To further validate the results of our previous analysis, qRT‐PCR was performed to detect TP73 mRNA expression in 53 glioma tissues. The results indicated that the expression level of TP73 was positively correlated with the grade of glioma (*p* = 0.0435) (Figure 6A), and the highest expressed in GBM subgroup. Survival analysis showed that TP73 high‐expression subgroup patients suffered worse OS (*p* = 0.1837) and PFS (*p* = 0.0448) than the low‐expression subgroup (Figure 6B–C), which was consistent with our previous analytical results. Although restricted by insufficient follow‐up and limited number of cases, the difference in OS between the two subgroups was not significant.

## 4 DISCUSSION

WHO Classification of Tumours of Central Nervous System (2016 version) introduced a series of molecular characteristics to refine based on the original histopathological classification.^3^ It is beneficial for neuro‐oncologists to choose an optimal adjuvant therapy individually and make a prognostic evaluation, based on these biomarkers. Emerging evidences support that molecular diagnosis exhibit better clinical guiding significance than traditional histopathological classification in WHO grade II/III glioma,^29^, ^30^ and our research also provides novel evidence for this perspective. Generally, the prognostic outcome of grade II/III glioma is superior to that of GBM, which makes it possible for us to evaluate the 3‐year, 5‐year, or long‐term survival of this type of glioma. Hence, more biomarkers with eminent specificity and sensitivity urgently need to be exploited to serve individualized medical care and evaluate the survival outcomes.

Currently, biomarkers, such as TERT, IDH mutation status, MGMT methylation and 1p/19q codeletion status, are widely applied in clinical practice. In 2008, Parsons DW^31^ discovered IDH1 mutation by analyzing a cohort of GBM samples’ WESeq data, and for the first time reported the association between IDH1 mutation and favorable OS. Subsequent study identified that IDH1/IDH2 mutations were more prevalent in WHO grade II/III glioma than GBM, and patients occurred IDH gene mutations share a better outcome than those of wildtype IDH genes.^32^ All those findings have long‐term significance in the evolution of molecular diagnosis of glioma. Our analysis found that TP73 alternation can be a risk factor in WHO grade II/III glioma.

In our study, integrated with TCGA and CGGA databases, we in‐depth analyzed the correlation between the level of TP73 gene mRNA expression, DNA methylation and the clinical outcomes of WHO grade II/III glioma. It was proposed that high TP73 expression can be a high‐risk factor for evaluating the survival outcome of patients diagnosed with WHO grade II/III glioma. And by combining with 1p/19q codeletion status, two variables can strongly strengthen the prognostic predictability, especially in 1p/19q intact subtype. Meanwhile, a cohort of glioma specimens was used for validation. The results indicated that the expression level of TP73 was positively correlated with the grade of glioma, and the high‐expression subgroup suffered a worse prognosis, which was consistent with previous analytical results. Finally, TP73‐related DEmRNAs enrichment analysis suggested that the abnormality of TP73 gene in WHO grade II/III glioma might involve cell cycle and p53 signaling pathway, which were closely related to the features of malignant phenotype.

Consistent with previous reports,^14^, ^33^ our genomewide analysis reconfirmed that TP73 gene rarely suffered mutation. But the mechanisms of the TP73 gene aberrance and the manifestation of significant correlation with survival outcome in WHO grade II/III glioma are still poorly understood. Therein, TP73 methylation status may be one of the factors. Additionally, we suspect that chromosome 1p/19q codeletion may also play a critical role in aberrant TP73 expression of WHO II/III glioma. Our analysis implicated that low expression of TP73 may partly because of gene dosage effect arising from 1p/19q codeletion in IDH‐mutant subtype. Conversely, the favorable prognostic outcome of 1p/19q codeletion glioma may essentially, partly due to the low expression of TP73. Although the prognostic prediction efficiency of TP73 in 1p/19q codeleted subtype was not so significant (Figure 4C). The possible reason may that there exists a strong correlation between two variables (TP73 expression and 1p deletion status). In contrast, TP73 profile is more valuable for evaluating the prognosis of IDH‐wildtype glioma patients (such patients always share 1p/19q intact). During our clinical practice, we encountered more glioma patients with 1p intact. Appending TP73 profile to evaluate the survival outcomes of such patients is also applicable and of greater significance. However, our study also has some limitations. First, due to two promoters in TP73 gene, different transcript variants may have different biological functions. And some of transcript variants’ functions are not completely understood, which may disturb our analysis. Second, the mechanism of TP73 and its isoforms contribute to the pathogenesis and progression of glioma is still poorly understood. We expect to make up for it in our future work.

In conclusion, we systematically analyzed the phenotypic characteristics of TP73 in WHO grade II/III glioma, and its correlation with survival time and clinical phenotypes (especially with molecular classification and tumor grade) through multi‐datasets. Our work showed the significant potential of TP73 gene as a molecular characteristic for evaluating the malignancy and survival outcome of WHO grade II/III glioma, which also provided a basis for later research on TP73 gene as a molecular target for glioma targeted therapy.

## CONFLICT OF INTEREST

The authors declared no conflict of interest.

## ETHICAL APPROVAL STATEMENT

This study was approved by the ethics committee of The Second Affiliated Hospital of Soochow University. All participants provided informed consent.

## Supporting information

Fig S1

Fig S2

Fig S3

Fig S4

Fig S5

Fig S6

Table S1

Table S2

Table S3

## Data Availability

All data used to support our findings in this study are available from the correspondent for reasonable request.
