# Supplementary figures and images for: Integrative analysis of TP73 profile prognostic significance in WHO grade II/III glioma
Source: Cancer Med. 2021 Jun 13;10(13):4644–57. doi: 10.1002/cam4.4016 (PMC8267133; doi:10.1002/cam4.4016)

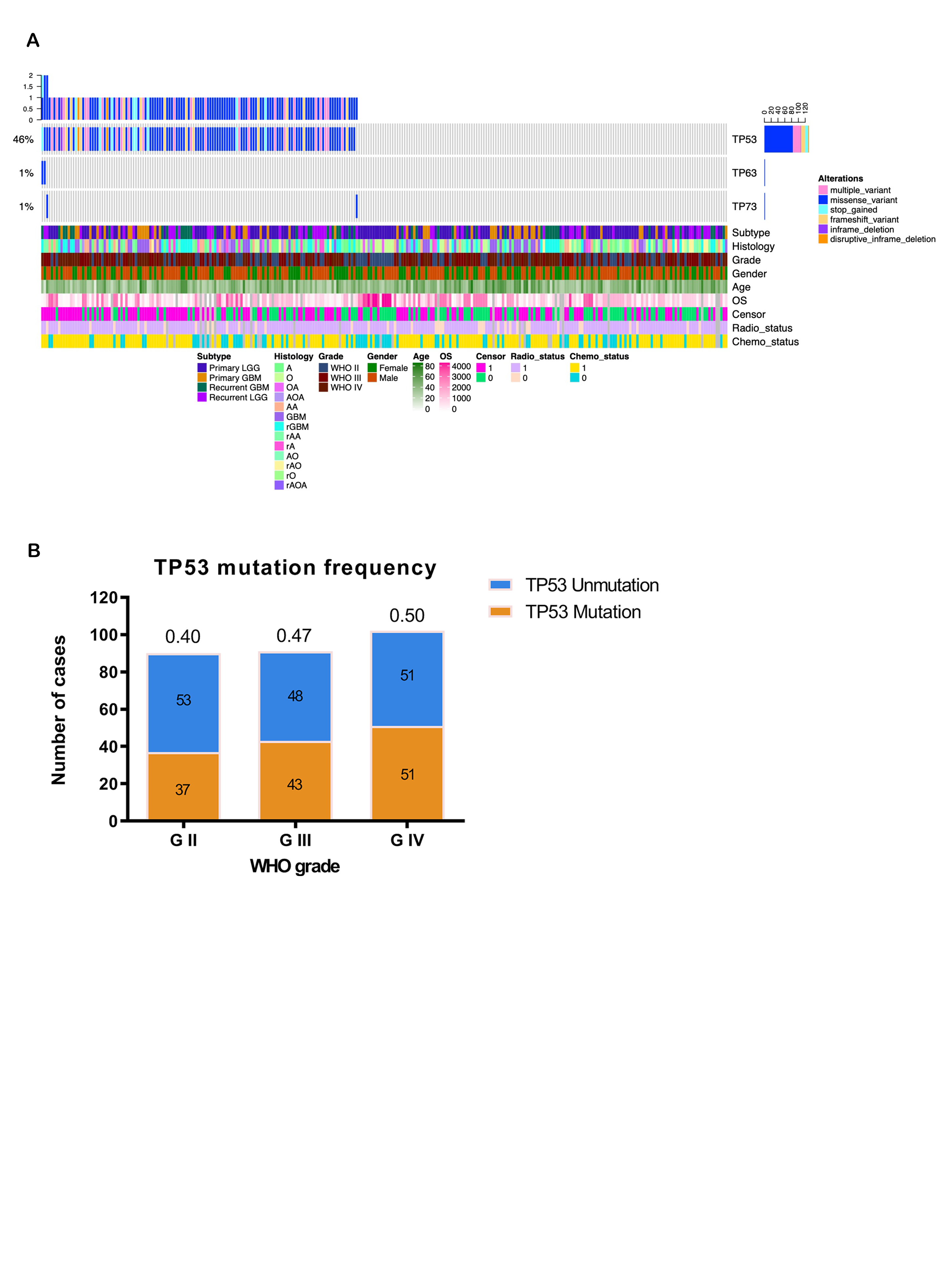

Supplement: Supplementary file 1 — Fig S1 [file CAM4-10-4644-s007.png]

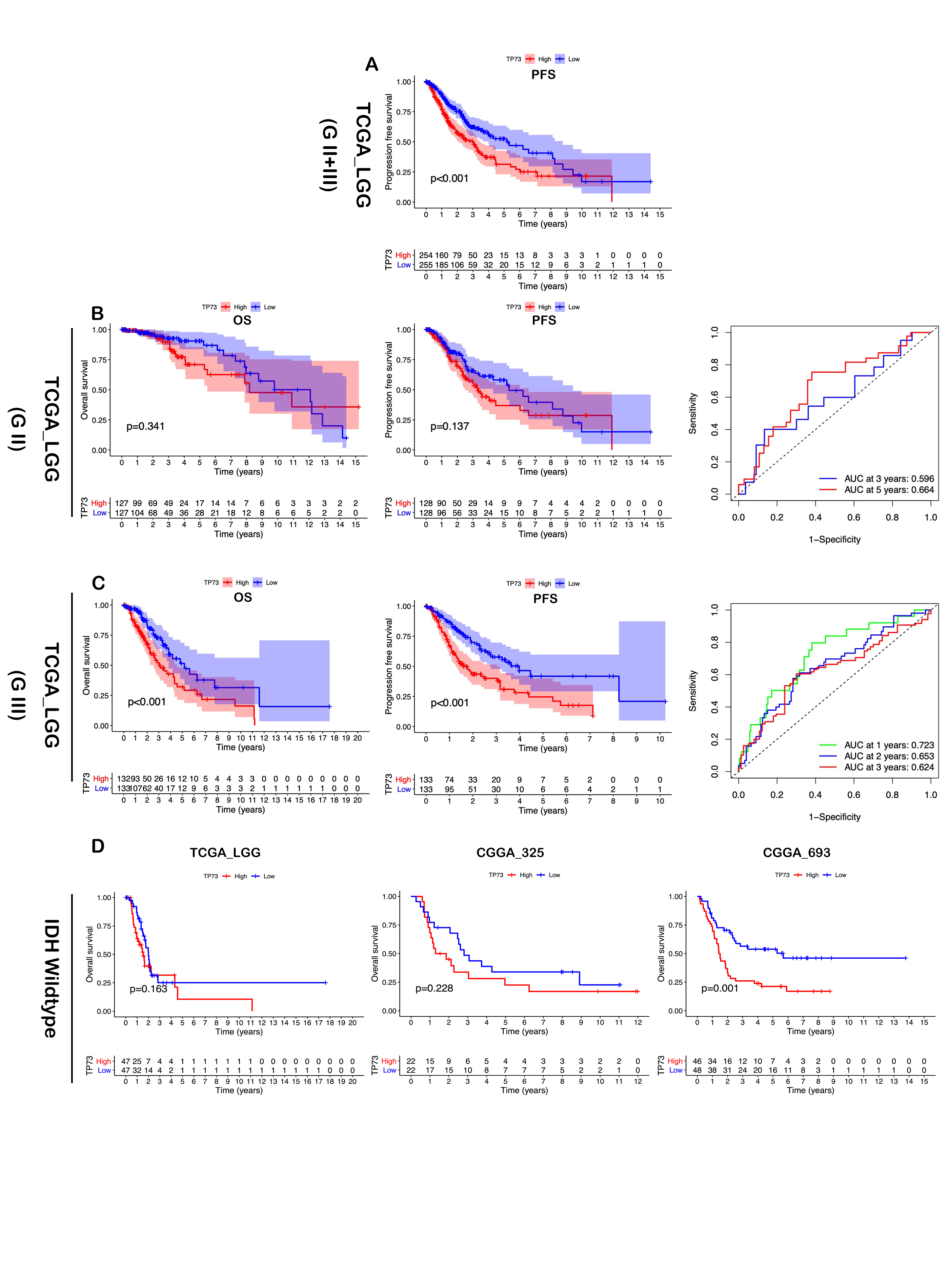

Supplement: Supplementary file 2 — Fig S2 [file CAM4-10-4644-s002.png]

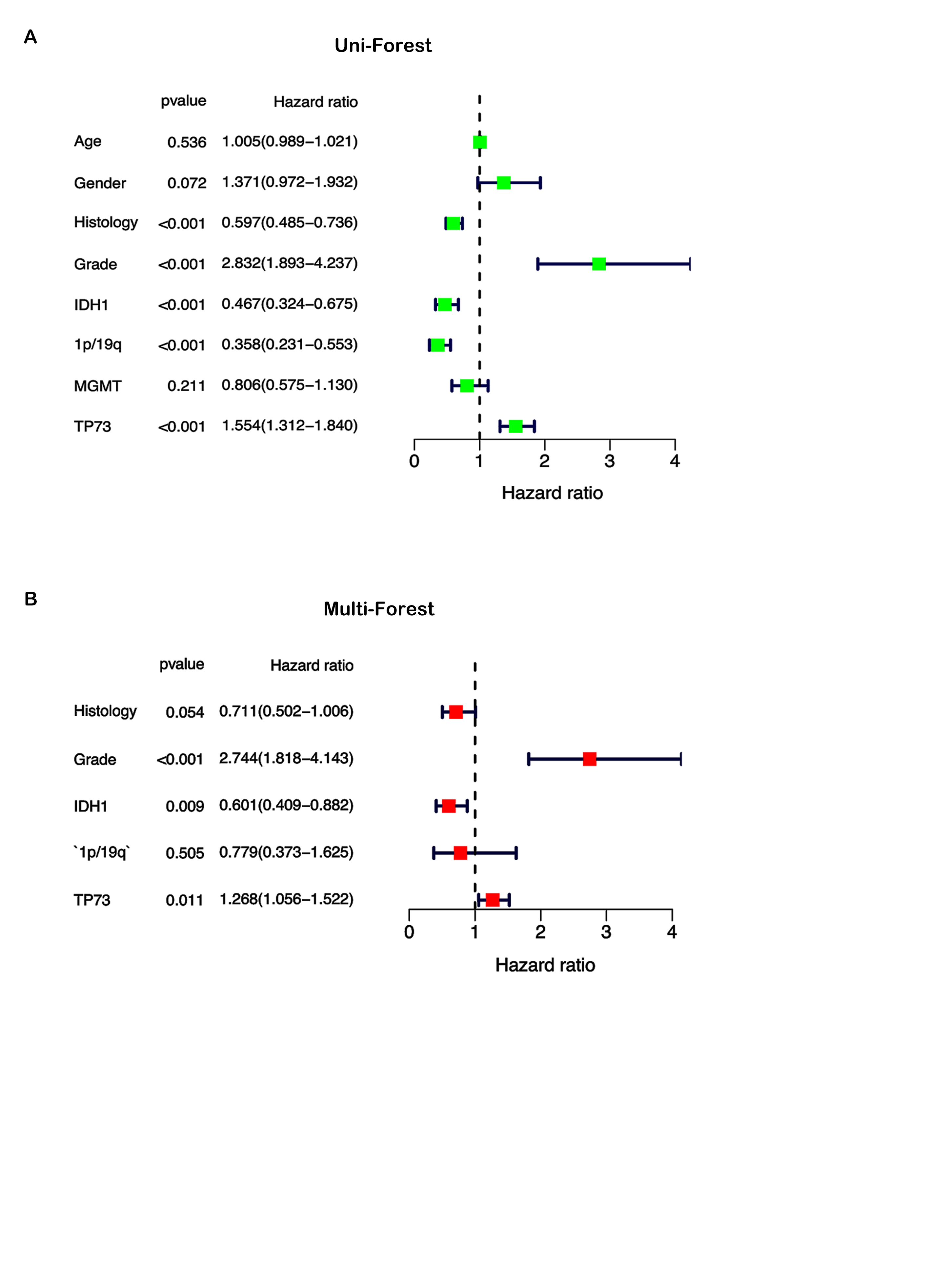

Supplement: Supplementary file 3 — Fig S3 [file CAM4-10-4644-s009.png]

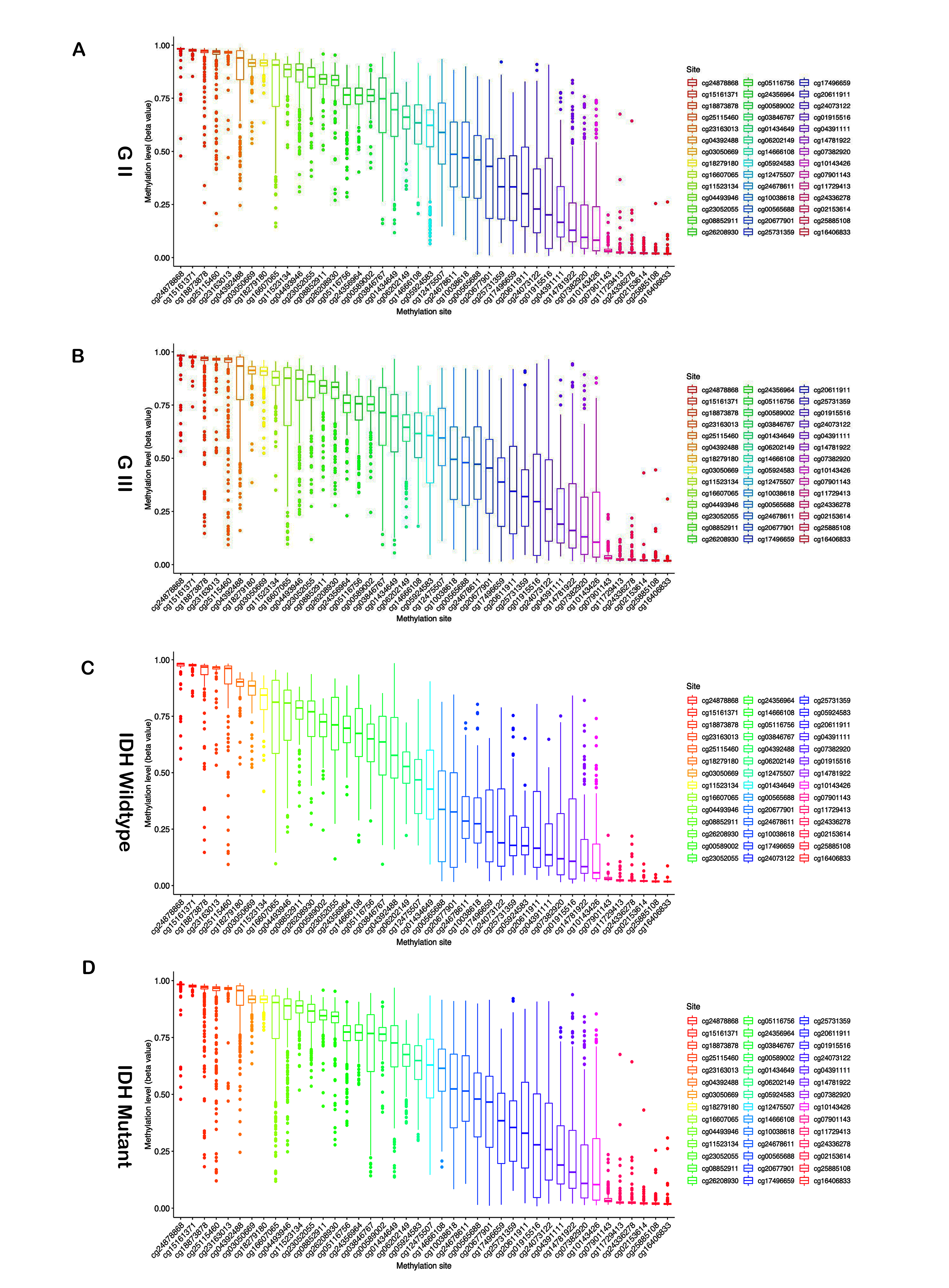

Supplement: Supplementary file 4 — Fig S4 [file CAM4-10-4644-s003.png]

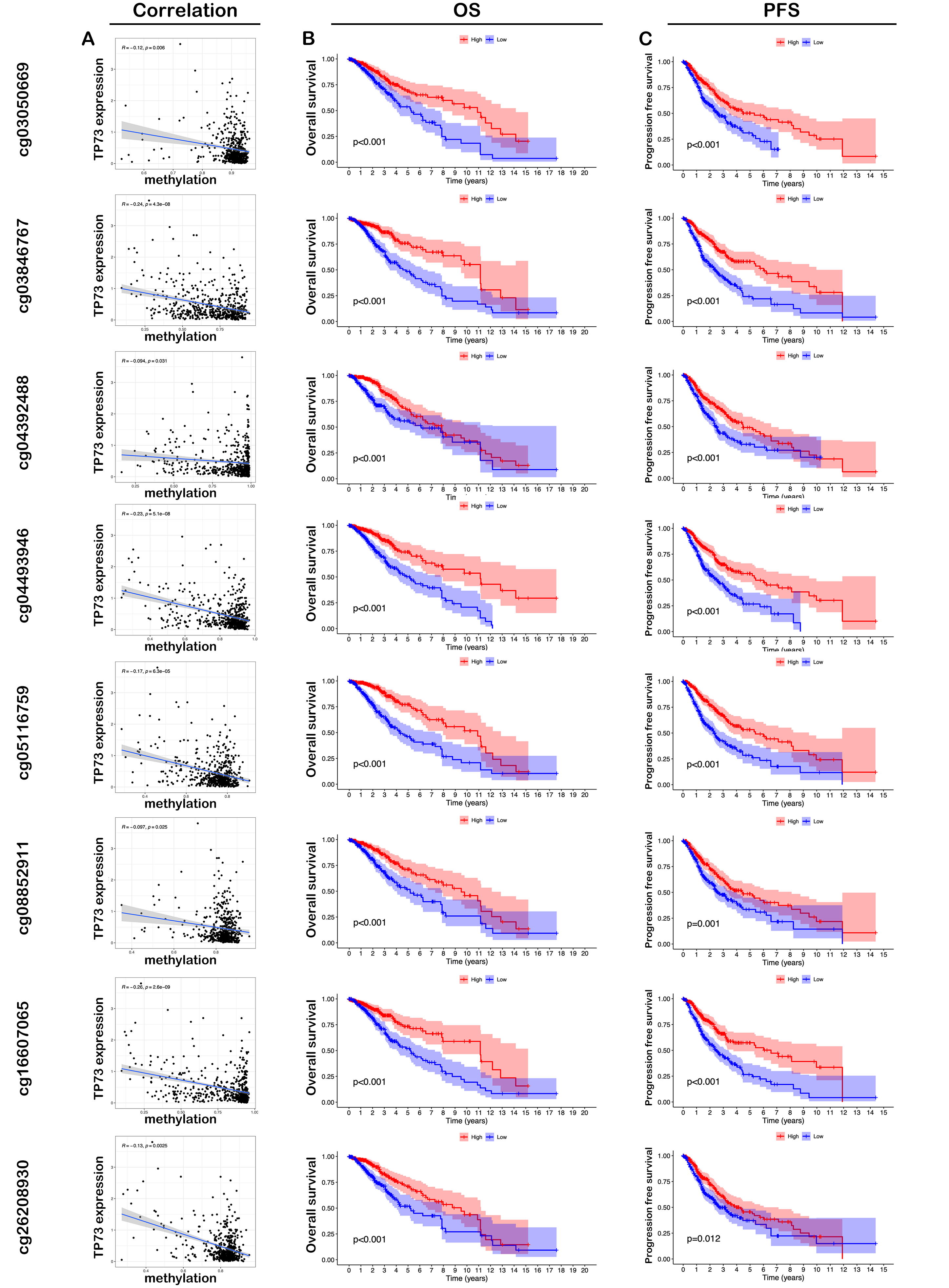

Supplement: Supplementary file 5 — Fig S5 [file CAM4-10-4644-s005.png]

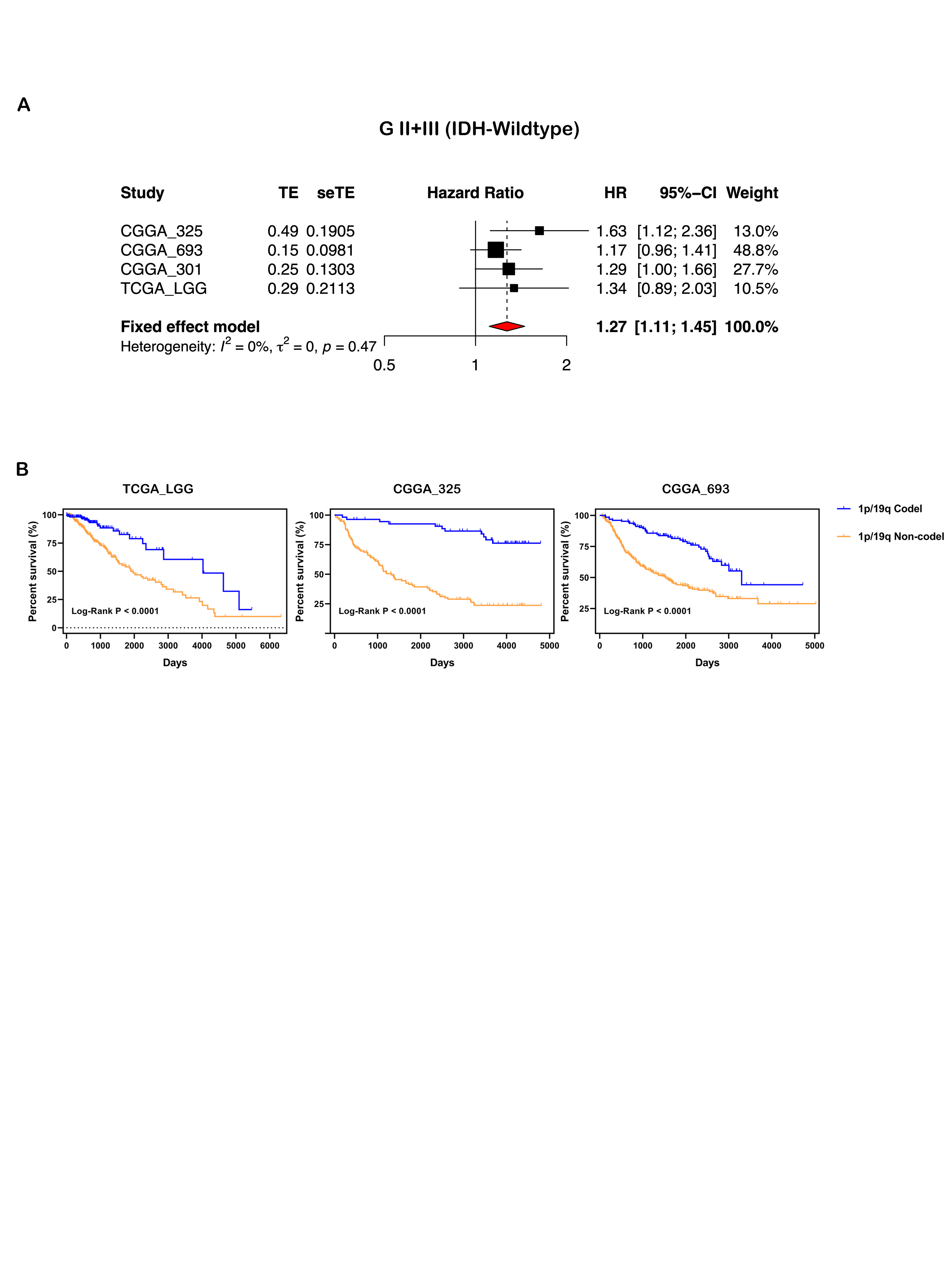

Supplement: Supplementary file 6 — Fig S6 [file CAM4-10-4644-s008.png]
