# Supplementary material for: Integrative analysis of TP73 profile prognostic significance in WHO grade II/III glioma
Source: Cancer Med. 2021 Jun 13;10(13):4644–57. doi: 10.1002/cam4.4016 (PMC8267133; doi:10.1002/cam4.4016)
Supplement: Supplementary file 7 — Table S1 [file CAM4-10-4644-s001.docx]

Table S1. The correlation analysis between TP73 methylation and clinical phenotypes with Chi-square test.

| **Covariates** | **Clinical Features** | | |  | | **TP73 methylation level** | | **P-value** |
| --- | --- | --- | --- | --- | --- | --- | --- | --- |
|  | **Sub-group** | **Percentage** | |  |  | **High** | **Low** |  |
| Age | <=65 | 421(93.35%) |  | | 208(92.44%) | | 213(94.25%) | 0.5623 |
|  | >65 | 30(6.65%) |  | | 17(7.56%) | | 13(5.75%) |  |
| Gender | Female | 199(44.12%) |  | | 97(43.11%) | | 102(45.13%) | 0.7357 |
|  | Male | 252(55.88%) |  | | 128(56.89%) | | 124(54.87%) |  |
| Histology | A | 167(37.03%) |  | | 69(30.67%) | | 98(43.36%) | 0.0203 |
|  | OA | 111(24.61%) |  | | 61(27.11%) | | 50(22.12%) |  |
|  | O | 173(38.36%) |  | | 95(42.22%) | | 78(34.51%) |  |
| Grade | II | 212(47.01%) |  | | 100(44.44%) | | 112(49.56%) | 0.2572 |
|  | III | 234(51.88%) |  | | 124(55.11%) | | 110(48.67%) |  |
|  | Unknow | 5(1.11%) |  | | 1(0.44%) | | 4(1.77%) |  |
| IDH Status | Mut | 363(80.49%) |  | | 214(95.11%) | | 149(65.93%) | < 0.0001 |
|  | Unknow | 2(0.44%) |  | | 1(0.44%) | | 1(0.44%) |  |
|  | WT | 86(19.07%) |  | | 10(4.44%) | | 76(33.63%) |  |
| 1p/19q Status | Codel | 151(33.48%) |  | | 103(45.78%) | | 48(21.24%) | < 0.0001 |
|  | Non-Codel | 300(66.52%) |  | | 122(54.22%) | | 178(78.76%) |  |
| Molecular Classification | IDH-Mut/Codel | 151(33.48%) |  | | 103(45.78%) | | 48(21.24%) | < 0.0001 |
|  | IDH-Mut/Non-codel | 212(47.01%) |  | | 111(49.33%) | | 101(44.69%) |  |
|  | IDH-WT | 86(19.07%) |  | | 10(4.44%) | | 76(33.63%) |  |
|  | Unknow | 2(0.44%) |  | | 1(0.44%) | | 1(0.44%) |  |
| MGMT Promoter | Methylated | 372(82.48%) |  | | 208(92.44%) | | 164(72.57%) | < 0.0001 |
|  | Unmethylated | 79(17.52%) |  | | 17(7.56%) | | 62(27.43%) |  |
| TERT Promoter | Mut | 130(28.82%) |  | | 68(30.22%) | | 62(27.43%) | 0.4793 |
|  | Unknow | 169(37.47%) |  | | 85(37.78%) | | 84(37.17%) |  |
|  | WT | 152(33.7%) |  | | 72(32%) | | 80(35.4%) |  |
| ATRX Status | Mut | 165(36.59%) |  | | 77(34.22%) | | 88(38.94%) | 0.3457 |
|  | Unknow | 2(0.44%) |  | | 1(0.44%) | | 1(0.44%) |  |
|  | WT | 284(62.97%) |  | | 147(65.33%) | | 137(60.62%) |  |

Abbreviations: NOS, Not Otherwise Specified; A, Astrocytoma; O, Oligodendroglioma; OA, Oligoastrocytoma; WT, Wildtype; Mut, Mutant; Codel, Codeletion.
