# Supplementary material for: Integrative analysis of TP73 profile prognostic significance in WHO grade II/III glioma
Source: Cancer Med. 2021 Jun 13;10(13):4644–57. doi: 10.1002/cam4.4016 (PMC8267133; doi:10.1002/cam4.4016)
Supplement: Supplementary file 8 — Table S2 [file CAM4-10-4644-s006.docx]

Table S2. The correlation analysis between TP73 expression and 1p/19q codeletion status in WHO grade II and grade III glioma, respectively.

| **Datasets** | **Grade** | **1p/19q codeletion Status** | | |  | **TP73 expression** | | **P-value** |
| --- | --- | --- | --- | --- | --- | --- | --- | --- |
|  |  | **Sub-group** | **Percentage** | |  | **High** | **Low** |  |
| CGGA_325 | II | Codel | | 44(43.14%) |  | 14(27.45%) | 30(58.82%) | 0.0027 |
|  |  | Non-Codel | | 58(56.86%) |  | 37(72.55%) | 21(41.18%) |  |
|  | III | Codel | | 16(20.51%) |  | 1(2.56%) | 15(38.46%) | 0.0003 |
|  |  | Non-Codel | | 62(79.49%) |  | 38(97.44%) | 24(61.54%) |  |
| CGGA_693 | II | Codel | | 58(34.12%) |  | 28(32.94%) | 30(35.29%) | 0.8715 |
|  |  | Non-Codel | | 112(65.88%) |  | 57(67.06%) | 55(64.71%) |  |
|  | III | Codel | | 73(31.2%) |  | 27(23.08%) | 46(39.32%) | 0.0111 |
|  |  | Non-Codel | | 161(68.8%) |  | 90(76.92%) | 71(60.68%) |  |

Abbreviations: Codel, Co-deletion; Non-Codel, Not Co-deletion.
