# Supplementary material for: Integrative analysis of TP73 profile prognostic significance in WHO grade II/III glioma
Source: Cancer Med. 2021 Jun 13;10(13):4644–57. doi: 10.1002/cam4.4016 (PMC8267133; doi:10.1002/cam4.4016)
Supplement: Supplementary file 9 — Table S3 [file CAM4-10-4644-s004.docx]

Table S3. TP73-related differentially expressed protein-coding mRNAs.

| **gene** | **conMean** | **treatMean** | **logFC** | **pValue** | **fdr** |
| --- | --- | --- | --- | --- | --- |
| NCAPG | 0.535635731 | 1.177371612 | 0.641735881 | 5.05E-24 | 3.96E-21 |
| PBK | 1.213088771 | 2.213653505 | 1.000564734 | 3.86E-23 | 1.77E-20 |
| SLC14A1 | 3.129309262 | 2.281300306 | -0.848008955 | 6.03E-08 | 8.42E-07 |
| IGFBP2 | 2.032640403 | 2.819373752 | 0.786733349 | 0.000166799 | 0.000728561 |
| TMSB15A | 1.987784848 | 2.746997609 | 0.759212761 | 9.67E-11 | 3.20E-09 |
| TOP2A | 1.562628863 | 2.803077671 | 1.240448808 | 1.75E-23 | 9.81E-21 |
| ASF1B | 1.000954326 | 1.833408156 | 0.83245383 | 1.20E-24 | 1.82E-21 |
| CENPF | 0.924710526 | 1.778351508 | 0.853640982 | 2.67E-23 | 1.34E-20 |
| CDC45 | 0.6693717 | 1.3093585 | 0.639986799 | 1.05E-21 | 2.58E-19 |
| TPX2 | 1.766315258 | 2.806890088 | 1.040574829 | 2.79E-24 | 2.49E-21 |
| DDN | 2.776372084 | 2.160004594 | -0.61636749 | 0.001787218 | 0.005342645 |
| SNAP25 | 5.730697201 | 5.105739001 | -0.6249582 | 0.000599535 | 0.002136711 |
| NDC80 | 0.633410188 | 1.318349977 | 0.684939789 | 1.07E-22 | 3.80E-20 |
| SFRP2 | 4.874048733 | 3.887503013 | -0.98654572 | 1.03E-06 | 9.72E-06 |
| SPC24 | 0.879767653 | 1.589781835 | 0.710014182 | 2.83E-20 | 5.10E-18 |
| GTSE1 | 0.650849321 | 1.303626515 | 0.652777194 | 1.16E-24 | 1.82E-21 |
| CHI3L1 | 2.813009014 | 3.637367988 | 0.824358974 | 0.007084676 | 0.016832951 |
| ZWINT | 1.825126593 | 2.588266964 | 0.763140372 | 5.73E-25 | 1.15E-21 |
| RRM2 | 0.905118518 | 1.815629979 | 0.91051146 | 2.18E-23 | 1.16E-20 |
| GRIN1 | 3.217792343 | 2.617032169 | -0.600760175 | 0.000386673 | 0.001481985 |
| TK1 | 1.354643641 | 2.126348491 | 0.77170485 | 4.53E-20 | 7.74E-18 |
| CDC20 | 1.218559411 | 2.139911865 | 0.921352455 | 2.03E-19 | 3.22E-17 |
| KIF4A | 0.804211047 | 1.532954445 | 0.728743399 | 2.98E-25 | 9.77E-22 |
| TACC3 | 1.389936152 | 2.05914297 | 0.669206817 | 1.21E-18 | 1.70E-16 |
| MKI67 | 0.866494981 | 1.699716536 | 0.833221555 | 2.03E-24 | 2.22E-21 |
| CCND1 | 4.022097874 | 4.622622208 | 0.600524335 | 1.00E-11 | 4.32E-10 |
| FCGBP | 1.994474663 | 2.607407993 | 0.612933331 | 1.54E-06 | 1.37E-05 |
| HJURP | 0.563472008 | 1.238984964 | 0.675512957 | 1.36E-24 | 1.90E-21 |
| MYBL2 | 1.020201981 | 2.068007374 | 1.047805393 | 1.35E-23 | 8.03E-21 |
| CKS2 | 3.264014164 | 3.970448226 | 0.706434062 | 4.28E-17 | 4.86E-15 |
| KIF2C | 0.906252653 | 1.673880037 | 0.767627384 | 2.09E-20 | 3.87E-18 |
| FANCI | 1.15203463 | 1.794669146 | 0.642634516 | 2.45E-24 | 2.30E-21 |
| CCNA2 | 1.057176873 | 1.766756105 | 0.709579231 | 1.68E-21 | 3.99E-19 |
| HPCAL4 | 3.860845698 | 3.202316903 | -0.658528795 | 6.42E-07 | 6.53E-06 |
| KIF11 | 1.261497154 | 1.899946204 | 0.638449051 | 8.30E-23 | 3.08E-20 |
| EZH2 | 1.664347844 | 2.374595358 | 0.710247514 | 1.37E-21 | 3.28E-19 |
| PHYHIP | 3.989168009 | 3.367323749 | -0.621844259 | 7.67E-05 | 0.000377654 |
| COL4A1 | 2.304694929 | 3.075089563 | 0.770394634 | 4.88E-09 | 9.52E-08 |
| DTL | 0.721313907 | 1.399056093 | 0.677742186 | 1.27E-25 | 6.61E-22 |
| UBE2C | 1.599454542 | 2.784626332 | 1.185171789 | 4.03E-22 | 1.10E-19 |
| CDCA5 | 1.402108691 | 2.115969334 | 0.713860643 | 3.68E-20 | 6.47E-18 |
| SLC17A7 | 3.511439902 | 2.874011461 | -0.637428441 | 0.001982999 | 0.005826095 |
| PACSIN1 | 3.092222541 | 2.434764865 | -0.657457676 | 0.001218899 | 0.0038764 |
| SH3GL2 | 5.108660164 | 4.437992675 | -0.670667489 | 3.67E-09 | 7.33E-08 |
| CAMK2A | 3.574656506 | 2.899081272 | -0.675575234 | 0.006649894 | 0.01597745 |
| MELK | 0.618004524 | 1.322329932 | 0.704325407 | 7.97E-24 | 5.06E-21 |
| FOXM1 | 1.342790396 | 2.33412519 | 0.991334794 | 5.56E-25 | 1.15E-21 |
| TYMS | 2.032035525 | 2.947141751 | 0.915106226 | 2.20E-22 | 6.35E-20 |
| GAS1 | 2.419716626 | 3.111828819 | 0.692112193 | 1.43E-17 | 1.71E-15 |
| TROAP | 0.528355657 | 1.165765115 | 0.637409458 | 1.41E-22 | 4.62E-20 |
| CDKN2C | 2.499661062 | 3.240970365 | 0.741309303 | 1.51E-11 | 6.21E-10 |
| CENPU | 0.951735812 | 1.728357815 | 0.776622003 | 1.47E-23 | 8.50E-21 |
| CDT1 | 1.368887223 | 1.978078558 | 0.609191335 | 1.85E-18 | 2.45E-16 |
| PTTG1 | 1.584930147 | 2.360956642 | 0.776026495 | 6.43E-18 | 8.21E-16 |
| CCNB2 | 0.878250947 | 1.657909493 | 0.779658546 | 1.15E-21 | 2.80E-19 |
| NCAPH | 0.835632561 | 1.533342 | 0.697709439 | 2.24E-25 | 8.80E-22 |
| TIMELESS | 1.871812568 | 2.487402409 | 0.615589841 | 2.01E-22 | 5.99E-20 |
| NUSAP1 | 1.756978347 | 2.858278182 | 1.101299835 | 5.63E-24 | 3.96E-21 |
| CDK1 | 1.08292329 | 1.880378342 | 0.797455052 | 1.94E-22 | 5.88E-20 |
| PIMREG | 1.04473995 | 1.982216202 | 0.937476253 | 4.30E-23 | 1.88E-20 |
| CDCA8 | 0.868122698 | 1.610437996 | 0.742315299 | 1.72E-22 | 5.36E-20 |
| NEFM | 2.53240513 | 1.920876833 | -0.611528296 | 0.001752974 | 0.005258655 |
| LMNB1 | 2.349879284 | 3.183907585 | 0.834028302 | 1.58E-24 | 1.96E-21 |
| BRINP1 | 3.81098293 | 3.115938418 | -0.695044512 | 3.66E-11 | 1.38E-09 |
| LINC01088 | 3.261417609 | 2.564219799 | -0.69719781 | 6.40E-07 | 6.52E-06 |
| KIFC1 | 1.215751681 | 2.147062498 | 0.931310817 | 3.38E-24 | 2.89E-21 |
| NUF2 | 0.774815801 | 1.400561856 | 0.625746054 | 4.25E-23 | 1.88E-20 |
| SNCG | 4.008716822 | 3.29532187 | -0.713394952 | 4.28E-07 | 4.63E-06 |
| BUB1 | 0.61173244 | 1.218030816 | 0.606298376 | 1.16E-22 | 4.00E-20 |
| UBE2T | 2.20307782 | 2.821138257 | 0.618060437 | 1.27E-13 | 8.32E-12 |
| SPC25 | 1.266229041 | 1.871114733 | 0.604885692 | 7.10E-16 | 6.78E-14 |
| ETNPPL | 5.33262332 | 4.417491735 | -0.915131585 | 1.27E-11 | 5.29E-10 |
| KIF20A | 0.542225131 | 1.199010041 | 0.656784911 | 1.98E-23 | 1.08E-20 |
| PTGDS | 7.850916012 | 7.142017754 | -0.708898258 | 4.47E-11 | 1.65E-09 |
| KIF18B | 1.114169437 | 1.833461051 | 0.719291615 | 1.54E-22 | 4.98E-20 |
| CRLF1 | 3.227190235 | 2.578818894 | -0.648371341 | 4.58E-06 | 3.43E-05 |
| GJB6 | 1.854427977 | 1.131162545 | -0.723265432 | 6.81E-07 | 6.83E-06 |
| MCM2 | 2.240706868 | 2.921546118 | 0.68083925 | 6.47E-23 | 2.55E-20 |
| SLC1A2 | 6.260612646 | 5.659582593 | -0.601030053 | 6.07E-07 | 6.24E-06 |
| CCK | 2.558994324 | 1.946491124 | -0.6125032 | 0.000267819 | 0.001086127 |
| VSNL1 | 3.517477417 | 2.79285812 | -0.724619297 | 0.000813388 | 0.002755603 |
| ALDOC | 8.492827777 | 7.865452929 | -0.627374848 | 2.91E-08 | 4.46E-07 |
| PRC1 | 1.666916888 | 2.334299327 | 0.667382439 | 7.95E-20 | 1.33E-17 |
| SELL | 4.230534099 | 3.595325332 | -0.635208767 | 0.000235453 | 0.000974555 |
| BIRC5 | 1.127668701 | 2.119390067 | 0.991721366 | 1.60E-22 | 5.09E-20 |
| USH1C | 3.05614724 | 2.416342673 | -0.639804567 | 2.58E-06 | 2.12E-05 |
| NTSR2 | 4.020589902 | 3.337455618 | -0.683134284 | 1.38E-06 | 1.25E-05 |
| GABRD | 3.612919298 | 2.899561762 | -0.713357536 | 1.29E-07 | 1.65E-06 |
| COL4A2 | 2.663994867 | 3.353542902 | 0.689548035 | 2.19E-08 | 3.49E-07 |
| SYT1 | 3.675847597 | 3.056337445 | -0.619510152 | 0.001686289 | 0.005082635 |
| E2F1 | 2.098850622 | 2.763809545 | 0.664958924 | 1.26E-16 | 1.33E-14 |
| AURKB | 0.792381198 | 1.612305433 | 0.819924235 | 9.56E-22 | 2.41E-19 |
| PDPN | 1.854885068 | 2.660602449 | 0.805717381 | 1.13E-05 | 7.40E-05 |
| HPSE2 | 2.592152483 | 1.807595899 | -0.784556585 | 1.23E-10 | 3.92E-09 |
| CCNB1 | 1.846456643 | 2.552973816 | 0.706517173 | 1.03E-21 | 2.56E-19 |
| METTL7B | 2.286769882 | 2.892461911 | 0.605692029 | 0.000203098 | 0.000862968 |
| NRGN | 5.527574514 | 4.916684639 | -0.610889876 | 0.007355135 | 0.017389446 |
| BUB1B | 0.64702469 | 1.34556934 | 0.69854465 | 3.09E-23 | 1.48E-20 |
| TP73 | 0.153936646 | 0.78217702 | 0.628240374 | 4.11E-88 | 8.09E-84 |
| TIMP1 | 3.295481264 | 3.94493117 | 0.649449906 | 0.005621676 | 0.013866002 |
